# Chebulagic Acid, a Hydrolyzable Tannin, Exhibited Antiviral Activity *in Vitro* and *in Vivo* against Human Enterovirus 71

**DOI:** 10.3390/ijms14059618

**Published:** 2013-05-03

**Authors:** Yajun Yang, Jinghui Xiu, Jiangning Liu, Li Zhang, Xiaoying Li, Yanfeng Xu, Chuan Qin, Lianfeng Zhang

**Affiliations:** 1 Key Laboratory of Human Diseases Comparative Medicine, Ministry of Health, Institute of Laboratory Animal Science, CAMS & Comparative Medicine Centre, PUMC, Beijing 100021, China; E-Mails: yangyajun484@hotmail.com (Y.Y.); xiujh@cnilas.org (J.X.); liujn@cnilas.org (J.L.); zhangl@cnilas.org (L.Z.); leexyhb@126.com (X.L.); 2 Key Laboratory of Human Diseases Animal Models, State administration of Traditional Chinese Medicine, Institute of Laboratory Animal Science, CAMS & Comparative Medicine Centre, PUMC, Beijing 100021, China; E-Mails: xuyanf@gmail.com (Y.X.); qinchuan@pumc.edu.cn (C.Q.)

**Keywords:** chebulagic acid, enterovirus 71, hydrolyzable tannin, antiviral activity

## Abstract

Human enterovirus 71 is one of the major causative agents of hand, foot and mouth disease in children under six years of age. Presently, no vaccines or antiviral drugs have been clinically available to employ against EV71. In this study, we demonstrate that treatment with chebulagic acid reduced the viral cytopathic effect on rhabdomyosarcoma cells with an IC_50_ of 12.5 μg/mL. The utilization of the chebulagic acid treatment on mice challenged with a lethal dose of enterovirus 71 was able to efficiently reduce mortality and relieve clinical symptoms through the inhibition of viral replication. Chebulagic acid may represent a potential therapeutic agent to control infections to enterovirus 71.

## 1. Introduction

Human enterovirus 71 (EV71) is a single-stranded positive-sense RNA virus belonging to the enterovirus genus of the Picornaviridae family [1]. EV71 infection was associated with a series of clinical diseases including hand, foot, and mouth disease (HFMD). Normally, this illness can be resolved spontaneously; however, EV71 infections can also cause severe neurological disorders that are a serious threat to children under 6 years of age [2]. Since it was first identified in 1969, outbreaks of infection due to this virus have been periodically reported worldwide, particularly in the Asia-Pacific region [3,4]. According to data from the Ministry of Health of the People’s Republic of China, EV71 infections have resulted in more than five million HFMD cases and more than two thousand deaths in China in the past five years. Unfortunately, there are no clinical vaccines or antiviral therapies currently available to prevent or treat infections due to EV71.

**Figure 1:**
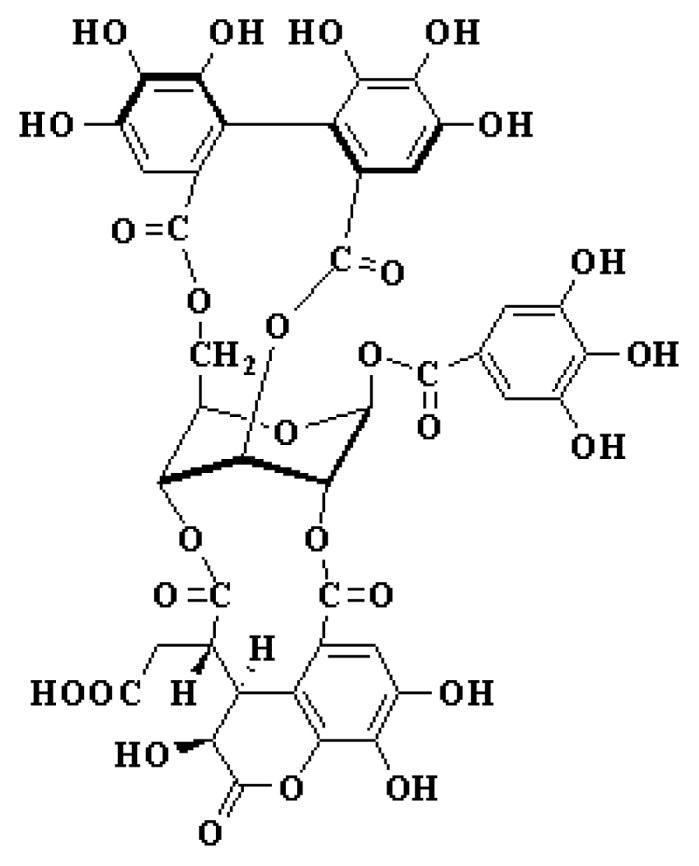
The structure of chebulagic acid.

Chebulagic acid (Figure 1), a plant-derived hydrolyzable tannin, was mainly isolated from the fruits of *Terminalia chebula*, which is a popular folk medicine in many Asian and African countries [5]. It is well known that many plants containing hydrolyzable tannins have been used for treating different diseases. In traditional Chinese medicine, the fruit of Terminalia chebula was commonly used for treating infectious diseases. Previous studies have shown that chebulagic acid has a wide range of pharmacological activities. More importantly, chebulagic acid has demonstrated the ability to inhibit hepatitis C virus [6], herpes simplex virus 1 [7], and human immunodeficiency virus [8]*in vitro*. Based on the antiviral activity of chebulagic acid, we explored its potency against EV71 in a preliminary screening assay, and the results showed that chebulagic acid was able to effectively inhibit EV71 infection. Herein, we reported the antiviral effect of chebulagic acid against EV71 both *in vitro* and *in vivo.*

## 2. Results and Discussion

### 2.1. Chebulagic Acid Inhibited EV71 Infection *in Vitro*

**Figure 2:**
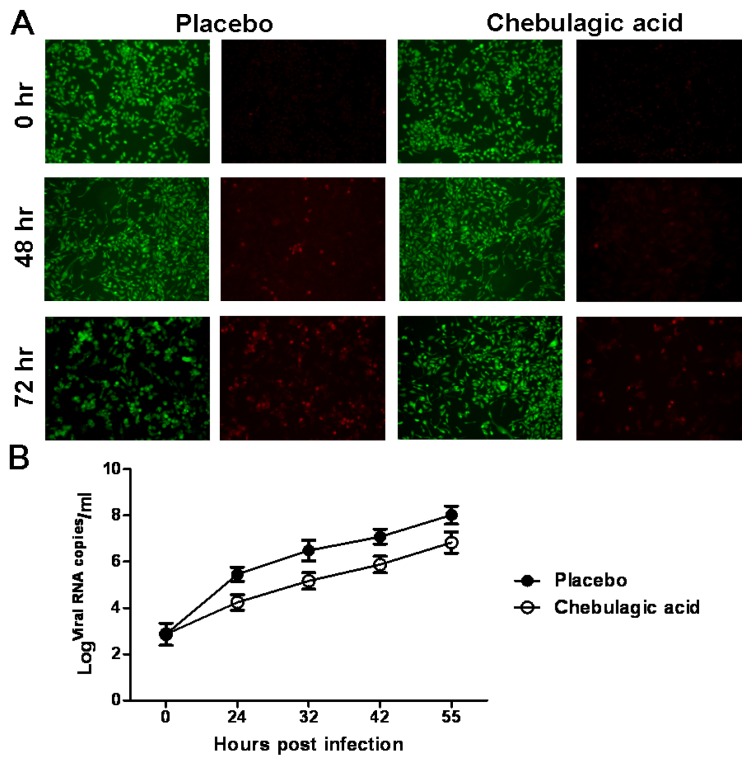
Effect of chebulagic acid on enterovirus 71 (EV71) replication in rhabdomyosarcoma (RD) cells. (**A**) The infected-RD cells were treated with chebulagic acid or saline at 2 h post EV71 infection, and then the cytopathic effect (CPE) of the RD cells were observed after AO/EB double staining under a light microscope (100×) at 0 h, 48 h and 72 h post infection, respectively; (**B**) The viral RNA copies in the culture supernatant of the RD cells were detected by quantitative RT-PCR (qRT-PCR). The data are expressed as the mean values of three independent experiments.

The efficacy of chebulagic acid was first tested on human rhabdomyosarcoma (RD) cells infected with EV71 in a plaque reduction assay. As a positive control, the IC_50_ value of ribavirin was 50 μg/mL. Chebulagic acid inhibited EV71 infection in a dose-dependent manner with an IC_50_ value of 12.5 μg/mL. Meanwhile, Chebulagic acid had a low cytotoxicity (CC_50_ > 200 μg/mL) in the RD cells. Chebulagic acid exerted sustained antiviral effects on the RD cells after AO/EB (acridine orange/ethidium bromide) double staining, as clearly shown in Figure 2A. The cytopathic effect of the RD cells caused by the viral infection was significantly inhibited after the treatment with chebulagic acid at 2 h post EV71 infection, and the viral RNA copies were also clearly reduced in the infected RD cells over time compared with that of the placebo control (Figure 2B).

### 2.2. Chebulagic Acid Reduced the Mortality of Mice upon Lethal EV71 Challenge

**Figure 3:**
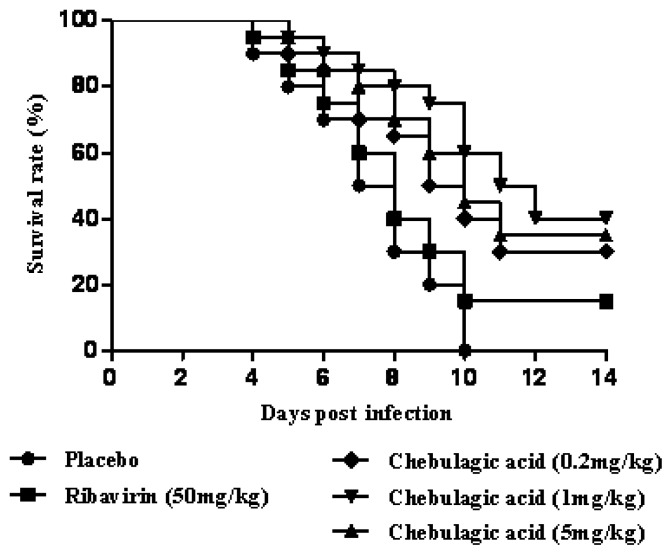
Chebulagic acid treatment reduced the mortality of EV71-infected mice. Survival rates of the EV71-infected mice treated with the placebo, ribavirin (50 mg/kg) and chebulagic acid (0.2, 1 and 5 mg/kg) were recorded at 14 dpi (*n* = 20).

Based on the effect of chebulagic acid *in vitro*, we confirmed its value by using the mouse model of the lethal EV71 infection. In this experiment, the placebo-treated mice developed paralysis at 3 dpi and all died within 10 dpi. In the ribavirin treatment group (50 mg/kg body weight), the survival rate of infected-mice was raised to 15% at 14 dpi. When the mice were treated with chebulagic acid at a dose of 0.2, 1 and 5 mg/kg, the survival time of the mice was greatly prolonged. It was also shown that 1 mg/kg was a proper dosage and that the survival rate was up to 40% at 14 dpi (Figure 3).

### 2.3. Chebulagic Acid Improved Symptoms of Infected-Mice by Inhibiting Viral Replication

**Figure 4:**
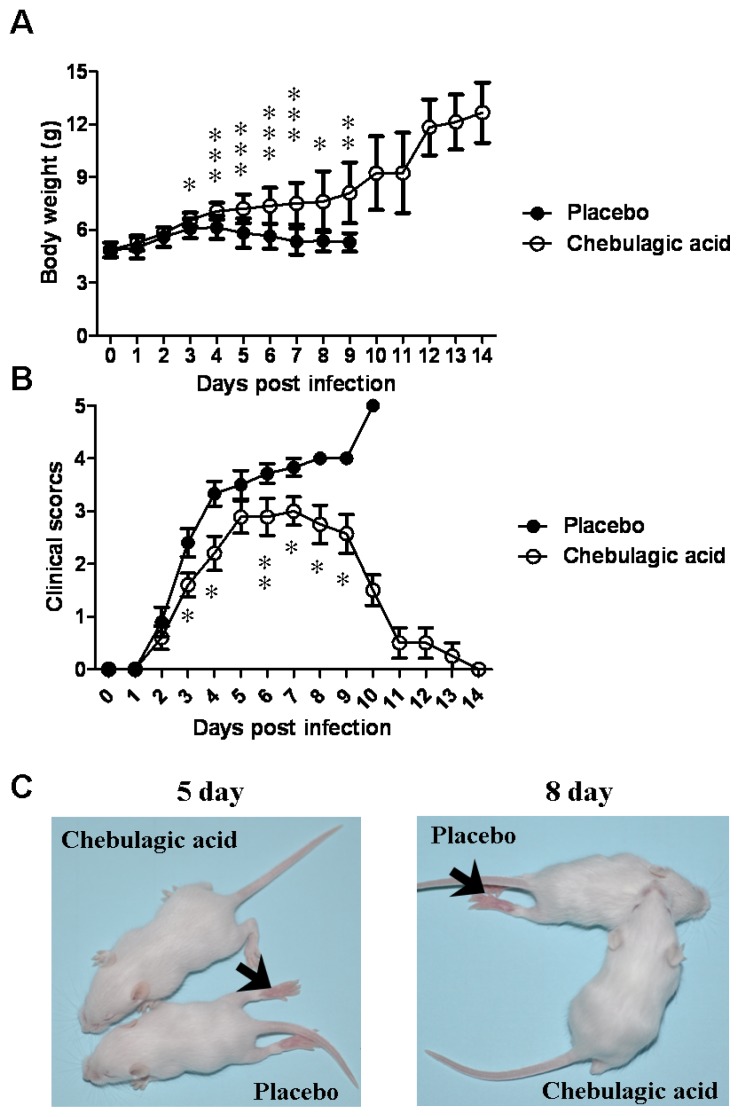
Chebulagic acid treatment relieved symptoms of EV71-infected mice. (**A**) The body weight of the infected mice treated with the placebo or chebulagic acid (1 mg/kg) was recorded in independent experiments (*n* = 20); (**B**) Clinical scores were systematically evaluated; (**C**) The typical phenotype of ruffled hair and paralysis of hind limbs caused by EV71 infection at 5 and 8 dpi (indicated by arrow) was shown, and the symptoms were prevented in the chebulagic acid treatment group (* *p* < 0.05, ** *p* < 0.01, *** *p* < 0.005).

**Figure 5:**
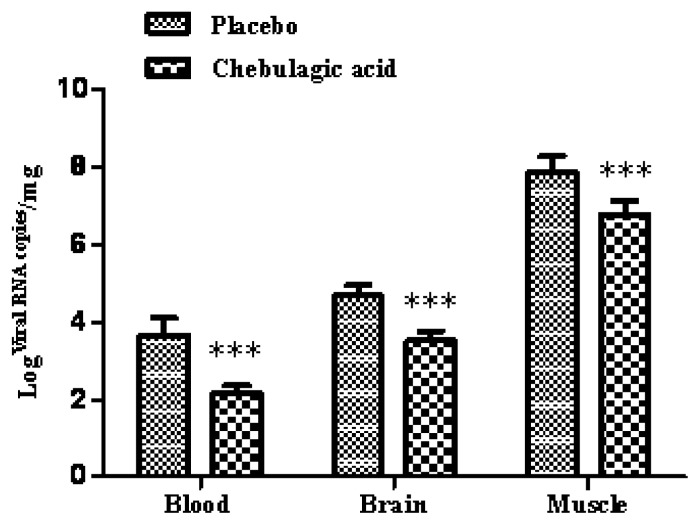
Chebulagic acid treatment inhibited the replication of EV71 in different tissues of the mice. The infected mice were treated with the placebo or with chebulagic acid at a dose of 1 mg/kg. The tissues were sampled and subjected to viral RNA copy analysis by qRT-PCR at 6 dpi, respectively (*n* = 8). The data are expressed as the mean values of three independent experiments (*** *p* < 0.005).

**Figure 6:**
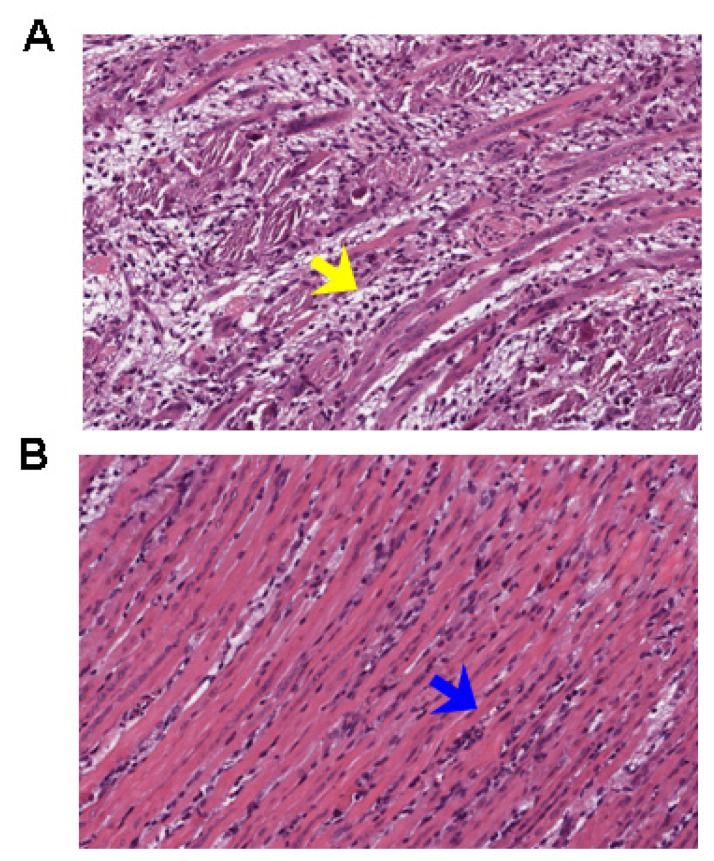
Chebulagic acid reduced pathological damage. The infected mice were treated with the placebo or chebulagic acid at a dose of 1 mg/kg. The pathological changes of muscle tissues at 9 dpi were observed after H & E staining. (**A**) The necrotising myositis was observed in the placebo-treated mice (the yellow arrow); (**B**) Moderate inflammation was observed in the muscle tissues of chebulagic acid-treated mice (the blue arrow). Magnification: 100×.

The body weight and clinical scores of the infected mice treated with the placebo or chebulagic acid (1 mg/kg) were systematically evaluated. As shown in Figure 4A, despite a slow increase in body weight for the first few days, mice in the chebulagic acid-treatment group began to recover on day 8 after challenge, whereas the weight of those in the placebo group continued to drop until death. Additionally, treatment with chebulagic acid noticeably reduced the clinical scores of the infected mice in comparison to those of the placebo (Figure 4B). Consistent with the results of the body weights and clinical scores, the symptoms of the EV71-infected mice were clearly prevented in the chebulagic acid-treatment group (Figure 4C). All of the surviving mice were recovered, and no evidence of disease was observed after 2 weeks. The significant recovery was due to the inhibition of viral replication in different tissues of the chebulagic acid-treated mice (Figure 5). Additionally, in contrast to the necrotising myositis in the placebo-treated mice, moderate inflammation was observed in the muscle tissues of the chebulagic acid-treated mice at 9 dpi (Figure 6).

Many natural plants, especially traditional medicinal herbs, are used in the treatment of viral infections. These traditional herbs are a plentiful source of antiviral agents. Several natural products have been found to display inhibitory activity on EV71, such as lycorine [9], matrine [10], ursolic acid [11], raoulic acid [12], epigallocatechin gallate [13], aloe-emodin [14], chrysosplenetin and penduletin [15]. We also reported that two hydrolyzable tannins, geraniin and punicalagin, exhibited a potent antiviral effect against EV71 *in vitro*. Moreover, they were able to reduce mortality in the infected mice by inhibiting viral replication [16,17]. Interestingly, another hydrolyzable tannin--punicalin did not demonstrate obvious antiviral efficacy. This founding indicated that the substituent in the 1-position of the glucose core is unnecessary, and different polyphenolic groups in other positions of glucose are key structural requrements for anti-EV71 activity. Further work is needed to resolve the detailed structure-activity relationship. In combination with the activity of chebulagic acid, these studies suggest that hydrolyzable tannins are an excellent source for antiviral discovery in the field of EV71 infection.

Chebulagic acid has demonstrated the ability to inhibit hepatitis C virus, herpes simplex virus 1, and human immunodeficiency virus *in vitro* [18]. Only the mechanism against HSV-1 was studied that chebulagic acid targeted and inactivated HSV-1 viral particles and could prevent binding, penetration, and cell-to-cell spread, as well as secondary infection. Chebulagic acid blocked interactions between cell surface glycosaminoglycans and HSV-1 glycoproteins [7]. It was estimated that the mechanism against EV-71 was related to the inhibition of viral absorption and/or penetration. According to other studies, Chebulagic acid also exhibited immunosuppressive effect on cytotocic T lymphocyte-mediated cytotocity [19]. Furthermore, chebulagic acid significantly suppressed the onset and progression of collagen-induced arthritis in mice via the induction of TGF-β and CD4^+^, CD25^+^ T cells [20]. These foundings indicated that chebulagic acid exhibited not only antiviral activity but also immunoregulation effect. We will investigate the mechanism of chebulagic acid against EV71 infection in detail in the further experiment.

## 3. Experimental Section

### 3.1. Cells, Viruses and Reagents

Human rhabdomyosarcoma (RD) cells were maintained in Dulbecco’s modified Eagle’s medium (DMEM) containing 10% foetal bovine serum (FBS). A clinically isolated EV71 strain FY0805 (GenBank accession No. HQ882182) and the mouse-adapted EV71 strain MP10 (GenBank accession No. HQ712020) derived from FY0805 were cultured in RD cells. The viral titres were determined using a plaque assay as described and working stocks of virus containing 10^9^ TCID_50_/mL were prepared for experiments. Chebulagic acid and ribavirin (purity > 97%) were purchased from the National Institute for the Control of Pharmaceutical and Biological Products (China).

### 3.2. Antiviral and Cytotoxicity Assay in RD Cells

For the antiviral assay, RD cells (2 × 10^4^ cells/well) were plated in 96-well plates with DMEM medium lacking antibiotics and were grown overnight to 90% confluence at 37 °C. The RD cells were then infected with 100 TCID_50_ of FY0805 and cultured continually in DMEM medium containing 2% FBS. The infected cells were treated with chebulagic acid in a set of concentrations in saline. The infected RD cells were observed for the cytopathic effect (CPE) after AO/EB (acridine orange/ethidium bromide) double staining or harvested at eight-hour intervals post infection to determine the number of viral RNA copies by quantitative RT-PCR (qRT-PCR). The half maximal inhibitory concentration (IC_50_) was defined as the concentration of chebulagic acid that caused a 50% CPE reduction compared to that of the saline-treated control. The concentration of chebulagic acid that was required for 50% cell cytotoxicity (CC_50_) was determined in the RD cells.

### 3.3. Mouse Protection Assay

Ten-day-old ICR mice were bred in an AAALAC-accredited facility and all of the animal protocols were approved by the Institutional Animal Care and Use Committee of the Institute of Laboratory Animal Science, Peking Union Medical College (GC-09-2077). For the lethal EV71 challenge, each 10-day-old mouse was intraperitoneally (i.p.) inoculated with 1 × 10^7^ TCID_50_ (lethal dose) of MP10. At 2 hours post infection, the infected mice were injected with different concentrations of chebulagic acid in saline daily twice for 5 days (i.p.). The placebo group was injected with the same volume of saline as the control group. The dose of chebulagic acid was selected based on previous reports. Ribavirin (50 mg/kg) is currently used as a positive control. The survival rates of the mice were monitored daily for 2 weeks.

In another separate experiment, each ten-day-old mouse was inoculated with 1 × 10^7^ TCID_50_ of MP10 as usual. At 2 h post infection, the infected mice were injected with chebulagic acid (1 mg/kg) or saline daily twice for 5 days (i.p.). The body weights and symptoms of infected and untreated mice were monitored daily for 2 weeks. The clinical scores were graded as follows: 0, healthy; 1, ruffled hair; 2, weakness in hind limbs; 3, paralysis in single hind limb; 4, paralysis in both hind limbs; and 5, death. After euthanasia, the blood, brain and muscle tissues of the mice were sampled at 6 dpi for virology analysis by qRT-PCR. Muscle tissues were sampled at 9 dpi for pathology analysis.

### 3.4. Determination of the Viral Load

qRT-PCR was used to detect the viral RNA copy number. Briefly, total RNA was isolated from cultured cells or tissues from the mice using the TRIzol reagent. The total RNA was then reverse transcribed using random hexamers with a reverse-transcription kit (Promega). The cDNA was subjected to quantitative PCR (QuantiTect SYBR Green RT-PCR kit, QIAGEN) with a Roche LightCycler 3.5 system for 40 cycles. The primers were EV71-S1 (5′-AGATAGGGTGGCAGATGTAATTGAAAG-3′) and EV71-A1 (5′-TAGCATTTGATGATGCTCCAATTTCAG-3′). A fragment corresponding to nucleotides 2462–2635 of FY0805 was adjusted to a concentration gradient (1 × 10^1^ to 1 × 10^8^ copies/μL) and was used as a standard to calculate the copy number of viral RNA.

### 3.5. Pathology

After euthanasia, the muscle tissues of the mice were immediately immersion-fixed in 10% buffered formalin for 48 h. The tissues were bisected, embedded in paraffin, and stained with hematoxylin and eosin stain (H & E). Ten sections of muscle were observed per animal in a blinded manner.

### 3.6. Statistics

All data are expressed as the mean ± S.D. The statistical significance of differences in mean values was assessed by Duncan’s multiple range test, following a one-way analysis of variance (ANOVA), and survival rates were analysed by Kaplan-Meier analysis. A *P*-value of <0.05 was considered to be significant.

## 4. Conclusions

In the present study, we evaluated the effect of chebulagic acid on EV71 *in vitro* and *in vivo*. To our knowledge, this is the first study that indicates the anti-EV71 effect of chebulagic acid. The antiviral properties of hydrolyzable tannins are well documented [18], but there is little information available in the literature about the activity of hydrolyzable tannins against EV71. Further research is needed to investigate the therapeutic effects and the structure-activity relationship of hydrolysable tannins in EV71 infection.
