# Correction: Yang et al. Chebulagic Acid, a Hydrolyzable Tannin, Exhibited Antiviral Activity *in Vitro* and *in Vivo* against Human Enterovirus 71. *Int. J. Mol. Sci.* 2013, *14*, 9618

**DOI:** 10.3390/ijms22168443

**Published:** 2021-08-06

**Authors:** Yajun Yang, Jinghui Xiu, Jiangning Liu, Li Zhang, Xiaoying Li, Yanfeng Xu, Chuan Qin, Lianfeng Zhang

**Affiliations:** 1 Key Laboratory of Human Diseases Comparative Medicine, Ministry of Health, Institute of Laboratory Animal Science, CAMS & Comparative Medicine Centre, PUMC, Beijing 100021, China; yangyajun484@hotmail.com (Y.Y.); xiujh@cnilas.org (J.X.); liujn@cnilas.org (J.L.); zhangl@cnilas.org (L.Z.); leexyhb@126.com (X.L.); 2 Key Laboratory of Human Diseases Animal Models, State administration of Traditional Chinese Medicine, Institute of Laboratory Animal Science, CAMS & Comparative Medicine Centre, PUMC, Beijing 100021, China; xuyanf@gmail.com (Y.X.); qinchuan@pumc.edu.cn (C.Q.)

The authors wish to make the following corrections to this paper [1]: in Figure 2A, the AO-EB cell staining picture in the placebo control group at 48 h is incorrect. Figure 2 should be replaced with the following figure.

The authors apologize for any inconvenience caused and state that the scientific conclusions are unaffected. The original article has been updated.

## Figures and Tables

**Figure 2:**
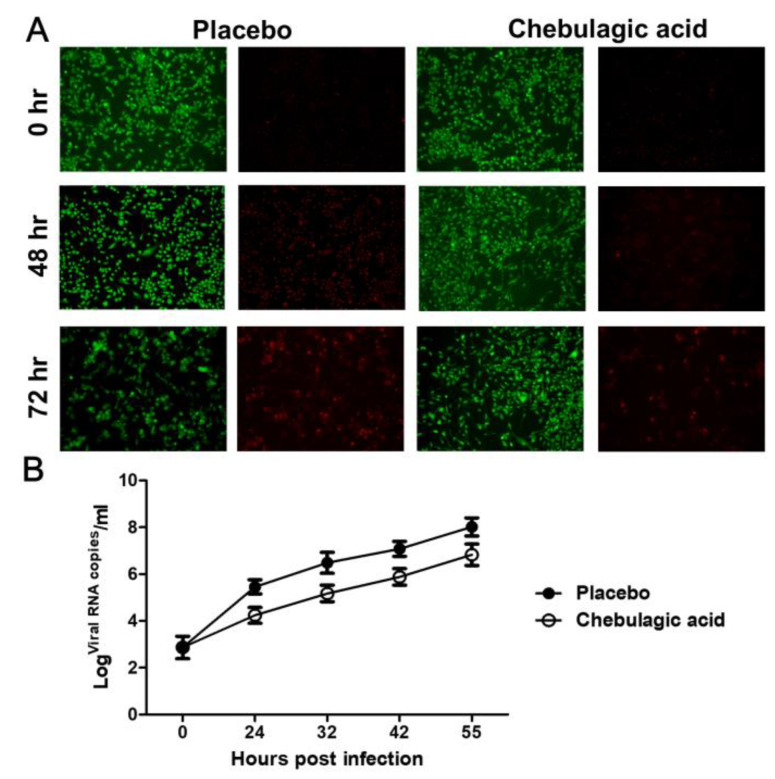
Effect of chebulagic acid on enterovirus 71 (EV71) replication in rhabdomyosarcoma (RD) cells. (**A**) The infected RD cells were treated with chebulagic acid or saline at 2 h post EV71 infection, and then the cytopathic effect (CPE) of the RD cells was observed after AO/EB double staining under a light microscope (100×) at 0 h, 48 h and 72 h post infection, respectively. (**B**) The viral RNA copies in the culture supernatant of the RD cells were detected by quantitative RT-PCR (qRT-PCR). The data are expressed as the mean values of three independent experiments.
